# *catena*-Poly[[(1,10-phenanthroline-κ^2^ *N*,*N*′)copper(I)]-μ-thio­cyanato-κ^2^ *N*:*S*]

**DOI:** 10.1107/S1600536810035002

**Published:** 2010-09-08

**Authors:** Hong Li, Shi Guo Zhang

**Affiliations:** a Binzhou Key Laboratory of Material Chemistry, Department of Chemistry and Chemical Engineering, Binzhou University, Binzhou 256603, People’s Republic of China

## Abstract

In the title complex, [Cu(NCS)(C_12_H_8_N_2_)]_*n*_, the Cu^I^ ion is in a distorted tetra­hedral CuN_3_S coordination geometry. The thio­cyanate ligand acts as bridging ligand, forming chains along [100]. A crystallographic mirror plane runs through the Cu^I^ ion, the thio­cyanate ligand and the middle of the phenanthroline ligand.

## Related literature

For related structures, see: Shi *et al.* (2006 ▶); Tadashi *et al.* (1990 ▶).
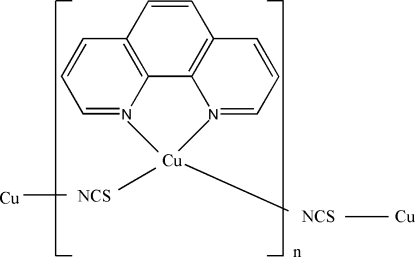


## Experimental

### 

#### Crystal data

[Cu(NCS)(C_12_H_8_N_2_)]
*M* *_r_* = 301.82
Orthorhombic, 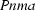

*a* = 7.9744 (15) Å
*b* = 11.948 (2) Å
*c* = 12.956 (2) Å
*V* = 1234.4 (4) Å^3^
*Z* = 4
Mo *K*α radiation
μ = 1.92 mm^−1^
*T* = 298 K
0.23 × 0.15 × 0.15 mm

#### Data collection

Bruker SMART APEX CCD diffractometer
Absorption correction: multi-scan (*SADABS*; Sheldrick, 1996 ▶) *T*_min_ = 0.667, *T*_max_ = 0.762
6226 measured reflections
1421 independent reflections
1146 reflections with *I* > 2σ(*I*)
*R*_int_ = 0.028

#### Refinement

*R*[*F* ^2^ > 2σ(*F* ^2^)] = 0.028
*wR*(*F* ^2^) = 0.080
*S* = 1.03
1421 reflections
89 parameters
H-atom parameters constrained
Δρ_max_ = 0.29 e Å^−3^
Δρ_min_ = −0.28 e Å^−3^

### 

Data collection: *SMART* (Bruker, 1997 ▶); cell refinement: *SAINT* (Bruker, 1997 ▶); data reduction: *SAINT*; program(s) used to solve structure: *SHELXTL* (Sheldrick, 2008 ▶); program(s) used to refine structure: *SHELXTL*; molecular graphics: *SHELXTL*; software used to prepare material for publication: *SHELXTL*.

## Supplementary Material

Crystal structure: contains datablocks I, global. DOI: 10.1107/S1600536810035002/lh5123sup1.cif

Structure factors: contains datablocks I. DOI: 10.1107/S1600536810035002/lh5123Isup2.hkl

Additional supplementary materials:  crystallographic information; 3D view; checkCIF report

## Figures and Tables

**Fig. 1.**
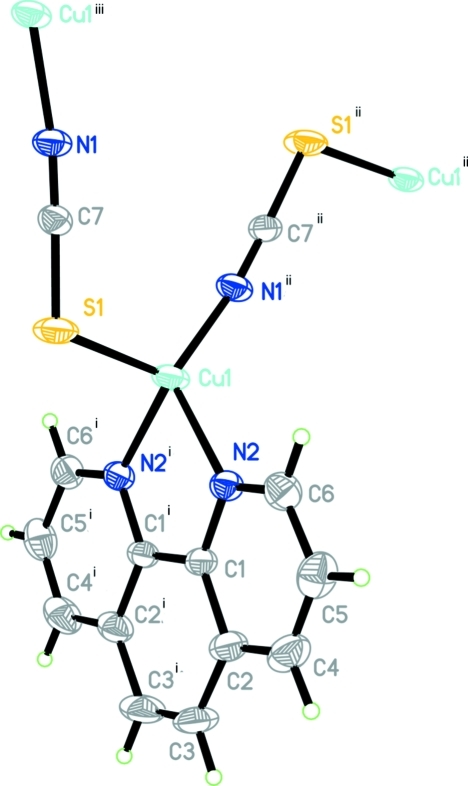
Part of the 1-D chain of the title compound with the atom-numbering scheme. Displacement ellipsoids are drawn at the 30% probability level. [symmetry codes: (i): x, -y + 3/2, z; (ii): x - 1/2, y, -z + 3/2; (iii): x + 1/2, y, -z + 3/2]

## supplementary crystallographic information

### Comment

1,10-phenanthroline and thiocyanate anions play an important role in modern coordination chemistry and many complexes have been published with them as ligands (e.g. Shi *et al.*, 2006; Tadashi *et al.* (1990). We originally tried to prepare a new divalent Cu(II) complex with these two ligands, but the title monovalent Cu(I) complex was fortuitously obtained. Herein we report its crystal structure.

Fig. 1 shows part of the title complex. The Cu^I^ ion is coordinated by three N atoms and one S atom, and is in a distorted tetrahedral coordination environment. The thiocyanate ligand acts as bridging forming a 1-D chain with a Cu···Cu distance of 5.9960 (9) Å.

### Experimental

A 5 ml H_2_O solution of Cu(ClO_4_)_2_6H_2_O (0.2000 g, 0.54 mmol) was added to a 10 ml methanol solution of 1,10-phenanthroline (0.1070 g, 0.54 mmol), and the mixture was stirred for a few minutes, then a 5 ml H_2_O solution of NaNCS (0.0875 g, 1.08 mmol) was added dropwise and the mixture was stirred for a few minutes and then placed in a Teflon-lined autoclave and heated at 433K for 144 h at autogenous pressure. After the contents of the autoclave were cooled to room temperature, the red single crystals were obtained.

### Refinement

All H atoms were placed in calculated positions and refined as riding with C—H = 0.93 Å, *U*_iso_ = 1.2*U*_eq_(C).

### Crystal data

**Table:**

[Cu(NCS)(C_12_H_8_N_2_)]	*F*(000) = 608
*M**_r_* = 301.82	*D*_x_ = 1.624 Mg m^−^^3^
Orthorhombic, *P**n**m**a*	Mo *K*α radiation, λ = 0.71073 Å
Hall symbol: -P 2ac 2n	Cell parameters from 2278 reflections
*a* = 7.9744 (15) Å	θ = 2.3–27.9°
*b* = 11.948 (2) Å	µ = 1.92 mm^−^^1^
*c* = 12.956 (2) Å	*T* = 298 K
*V* = 1234.4 (4) Å^3^	Block, red
*Z* = 4	0.23 × 0.15 × 0.15 mm

### Data collection

**Table:**

Bruker SMART APEX CCD diffractometer	1421 independent reflections
Radiation source: fine-focus sealed tube	1146 reflections with *I* > 2σ(*I*)
graphite	*R*_int_ = 0.028
φ and ω scans	θ_max_ = 27.0°, θ_min_ = 2.3°
Absorption correction: multi-scan (*SADABS*; Sheldrick, 1996)	*h* = −9→10
*T*_min_ = 0.667, *T*_max_ = 0.762	*k* = −15→15
6226 measured reflections	*l* = −5→16

### Refinement

**Table:**

Refinement on *F*^2^	Secondary atom site location: difference Fourier map
Least-squares matrix: full	Hydrogen site location: inferred from neighbouring sites
*R*[*F*^2^ > 2σ(*F*^2^)] = 0.028	H-atom parameters constrained
*wR*(*F*^2^) = 0.080	*w* = 1/[σ^2^(*F*_o_^2^) + (0.0508*P*)^2^] where *P* = (*F*_o_^2^ + 2*F*_c_^2^)/3
*S* = 1.03	(Δ/σ)_max_ = 0.001
1421 reflections	Δρ_max_ = 0.29 e Å^−^^3^
89 parameters	Δρ_min_ = −0.28 e Å^−^^3^
0 restraints	Extinction correction: *SHELXTL* (Sheldrick, 2008), Fc^*^=kFc[1+0.001xFc^2^λ^3^/sin(2θ)]^-1/4^
Primary atom site location: structure-invariant direct methods	Extinction coefficient: 0.0039 (10)

### Special details

**Table:**

Geometry. All e.s.d.'s (except the e.s.d. in the dihedral angle between two l.s. planes) are estimated using the full covariance matrix. The cell e.s.d.'s are taken into account individually in the estimation of e.s.d.'s in distances, angles and torsion angles; correlations between e.s.d.'s in cell parameters are only used when they are defined by crystal symmetry. An approximate (isotropic) treatment of cell e.s.d.'s is used for estimating e.s.d.'s involving l.s. planes.
Refinement. Refinement of *F*^2^ against ALL reflections. The weighted *R*-factor *wR* and goodness of fit *S* are based on *F*^2^, conventional *R*-factors *R* are based on *F*, with *F* set to zero for negative *F*^2^. The threshold expression of *F*^2^ > σ(*F*^2^) is used only for calculating *R*-factors(gt) *etc*. and is not relevant to the choice of reflections for refinement. *R*-factors based on *F*^2^ are statistically about twice as large as those based on *F*, and *R*- factors based on ALL data will be even larger.

### Fractional atomic coordinates and isotropic or equivalent isotropic displacement parameters (Å^2^)

**Table:**

	*x*	*y*	*z*	*U*_iso_*/*U*_eq_	
C1	0.13936 (17)	0.68993 (15)	1.13076 (12)	0.0406 (4)	
C2	0.1011 (2)	0.63175 (18)	1.22243 (13)	0.0543 (5)	
C3	0.0628 (3)	0.69444 (18)	1.31338 (13)	0.0712 (6)	
H3	0.0372	0.6565	1.3740	0.085*	
C4	0.1051 (2)	0.51479 (18)	1.21873 (16)	0.0669 (6)	
H4	0.0792	0.4731	1.2772	0.080*	
C5	0.1468 (2)	0.46262 (19)	1.12958 (17)	0.0648 (6)	
H5	0.1491	0.3849	1.1262	0.078*	
C6	0.1862 (2)	0.52657 (16)	1.04275 (16)	0.0528 (5)	
H6	0.2175	0.4895	0.9826	0.063*	
C7	0.5834 (3)	0.7500	0.79202 (17)	0.0436 (5)	
Cu1	0.23713 (4)	0.7500	0.92283 (2)	0.04865 (16)	
N1	0.6286 (2)	0.7500	0.70801 (14)	0.0487 (5)	
N2	0.18111 (17)	0.63783 (12)	1.04164 (11)	0.0412 (3)	
S1	0.52660 (9)	0.7500	0.91372 (4)	0.0702 (3)	

### Atomic displacement parameters (Å^2^)

**Table:**

	*U*^11^	*U*^22^	*U*^33^	*U*^12^	*U*^13^	*U*^23^
C1	0.0342 (8)	0.0577 (10)	0.0300 (8)	−0.0010 (7)	0.0005 (6)	0.0033 (7)
C2	0.0467 (10)	0.0768 (13)	0.0392 (9)	−0.0047 (9)	0.0023 (8)	0.0131 (9)
C3	0.0708 (12)	0.1093 (18)	0.0335 (9)	−0.0073 (11)	0.0146 (9)	0.0111 (9)
C4	0.0664 (14)	0.0748 (15)	0.0593 (13)	−0.0081 (10)	−0.0013 (10)	0.0283 (11)
C5	0.0638 (13)	0.0510 (11)	0.0796 (16)	−0.0007 (10)	−0.0080 (12)	0.0169 (11)
C6	0.0548 (11)	0.0519 (11)	0.0517 (11)	0.0028 (9)	−0.0052 (9)	−0.0009 (9)
C7	0.0417 (12)	0.0567 (15)	0.0323 (12)	0.000	−0.0043 (10)	0.000
Cu1	0.0589 (3)	0.0631 (3)	0.0240 (2)	0.000	0.00043 (12)	0.000
N1	0.0563 (13)	0.0619 (13)	0.0278 (9)	0.000	0.0038 (9)	0.000
N2	0.0423 (7)	0.0469 (8)	0.0345 (7)	0.0000 (6)	−0.0012 (6)	0.0021 (6)
S1	0.0499 (4)	0.1360 (8)	0.0245 (3)	0.000	0.0024 (3)	0.000

### Geometric parameters (Å, °)

**Table:**

C1—N2	1.353 (2)	C5—H5	0.9300
C1—C2	1.410 (2)	C6—N2	1.330 (2)
C1—C1^i^	1.435 (4)	C6—H6	0.9300
C2—C4	1.399 (3)	C7—N1	1.147 (3)
C2—C3	1.429 (2)	C7—S1	1.640 (2)
C3—C3^i^	1.328 (4)	Cu1—N1^ii^	1.9033 (19)
C3—H3	0.9300	Cu1—N2^i^	2.0893 (14)
C4—C5	1.354 (3)	Cu1—N2	2.0893 (14)
C4—H4	0.9300	Cu1—S1	2.3113 (9)
C5—C6	1.396 (3)	N1—Cu1^iii^	1.9033 (19)
			
N2—C1—C2	123.04 (17)	N2—C6—C5	123.3 (2)
N2—C1—C1^i^	117.39 (9)	N2—C6—H6	118.4
C2—C1—C1^i^	119.55 (11)	C5—C6—H6	118.4
C4—C2—C1	117.32 (19)	N1—C7—S1	177.7 (2)
C4—C2—C3	123.83 (19)	N1^ii^—Cu1—N2^i^	123.98 (6)
C1—C2—C3	118.84 (18)	N1^ii^—Cu1—N2	123.98 (6)
C3^i^—C3—C2	121.60 (11)	N2^i^—Cu1—N2	79.80 (8)
C3^i^—C3—H3	119.2	N1^ii^—Cu1—S1	114.12 (6)
C2—C3—H3	119.2	N2^i^—Cu1—S1	104.55 (4)
C5—C4—C2	119.65 (19)	N2—Cu1—S1	104.55 (4)
C5—C4—H4	120.2	C7—N1—Cu1^iii^	171.3 (2)
C2—C4—H4	120.2	C6—N2—C1	117.26 (16)
C4—C5—C6	119.4 (2)	C6—N2—Cu1	129.99 (13)
C4—C5—H5	120.3	C1—N2—Cu1	112.71 (11)
C6—C5—H5	120.3	C7—S1—Cu1	108.96 (9)
			
N2—C1—C2—C4	−0.9 (2)	C1^i^—C1—N2—C6	177.93 (12)
C1^i^—C1—C2—C4	−179.14 (12)	C2—C1—N2—Cu1	−178.16 (12)
N2—C1—C2—C3	178.35 (16)	C1^i^—C1—N2—Cu1	0.08 (11)
C1^i^—C1—C2—C3	0.1 (2)	N1^ii^—Cu1—N2—C6	58.10 (18)
C4—C2—C3—C3^i^	179.09 (13)	N2^i^—Cu1—N2—C6	−177.60 (14)
C1—C2—C3—C3^i^	−0.2 (2)	S1—Cu1—N2—C6	−75.07 (16)
C1—C2—C4—C5	0.9 (3)	N1^ii^—Cu1—N2—C1	−124.40 (10)
C3—C2—C4—C5	−178.38 (19)	N2^i^—Cu1—N2—C1	−0.09 (13)
C2—C4—C5—C6	0.4 (3)	S1—Cu1—N2—C1	102.44 (10)
C4—C5—C6—N2	−1.7 (3)	N1—C7—S1—Cu1	180.00 (2)
S1—C7—N1—Cu1^iii^	0.00 (2)	N1^ii^—Cu1—S1—C7	0.0
C5—C6—N2—C1	1.7 (3)	N2^i^—Cu1—S1—C7	−138.50 (4)
C5—C6—N2—Cu1	179.08 (13)	N2—Cu1—S1—C7	138.50 (4)
C2—C1—N2—C6	−0.3 (2)		

Symmetry codes: (i) *x*, −*y*+3/2, *z*; (ii) *x*−1/2, *y*, −*z*+3/2; (iii) *x*+1/2, *y*, −*z*+3/2.
